# Fall Prevention in Dutch Physician Practices: A Descriptive Study

**DOI:** 10.1093/geroni/igaa057.758

**Published:** 2020-12-16

**Authors:** Wytske Meekes, C J Leemrijse, J C Korevaar, L A M van de Goor

**Affiliations:** 1 Tilburg University, Tilburg, Noord-Brabant, Netherlands; 2 Nivel (Netherlands institute for health services research), Utrecht, Utrecht, Netherlands

## Abstract

Falls are an important health threat among frail older people. Physicians are often the first to contact for health issues and can be seen as designated professionals to provide fall prevention. However, it is unknown what they exactly do and why regarding fall prevention. This study aims to describe what physicians in the Netherlands do during daily practice in regards to fall prevention. About 65 physicians (34 practices) located throughout the Netherlands were followed up for 12 months. When a physician entered specific ICPC-codes related to frailty and falls in the Hospital Information System, the physician received a pop-up asking if the patient is frail. If so, the physician subsequently completed a questionnaire. The physicians completed 1396 questionnaires. More than half (n=726) of the patients had experienced a fall in the previous year and/or had a fear of falling (FOF) and 37% of these patients received fall prevention. Physicians did not know of 20% of the patients if they had experienced a fall and of 29% of the patients if they had a FOF. The three most often treated underlying causes were mobility problems, FOF and cardiovascular risk factors. The results show that physicians are not always aware of a patient’s fall history and/or FOF and that only part of these patients receives fall prevention. Hence, it might be important to develop and implement strategies for systematic fall risk screening and fall prevention provision in the primary care setting to reduce falls among frail older people.

